# isMap – immunological synapse map analysis program

**DOI:** 10.3389/fimmu.2026.1746651

**Published:** 2026-03-04

**Authors:** Amanda Holstad Singleton, Anthony Manet, Salvatore Valvo, Marike Feenstra, Harald Stenmark, Michael L. Dustin, Audun Kvalvaag

**Affiliations:** 1 Department of Molecular Cell Biology, Institute for Cancer Research, Oslo University Hospital, Oslo, Norway; 2 Centre for Cancer Cell Reprogramming, Institute of Clinical Medicine, Faculty of Medicine, University of Oslo, Oslo, Norway; 3 Institute for Cancer Genetics and Informatics, Oslo University Hospital, Oslo, Norway; 4 Nuffield Department of Orthopedics, Rheumatology and Musculoskeletal Sciences, Kennedy Institute of Rheumatology, Oxford, United Kingdom; 5 Nuffield Department of Medicine, Chinese Academy of Medical Sciences Oxford Institute, University of Oxford, Oxford, United Kingdom

**Keywords:** PD-L1, CD58, CD8+ T cells, CD80, CD86, ICAM-1, ICOSL, immunological synapse

## Abstract

T cell activation is initiated when T cells recognize their cognate antigen on the surface of antigen presenting cells (APCs). This triggers formation of a specialized membrane interface termed the immunological synapse (IS) which governs the spatial organization of the intercellular protein interactions ultimately determining the T cell response. While this is a fundamental process in adaptive immunity, tools for quantitative analysis and visualization of the IS molecular architecture are lacking. Here we present isMap, a computational framework for automated cell segmentation and quantification of various parameters related to T cell activation and IS formation on supported lipid bilayers (SLBs), including fluorescence intensity measurements, colocalization analysis and radial averaging. We validate isMap by confirming previous results showing that CD58 initially clusters with T cell receptor (TCR) before segregating into a distal ring during synapse maturation in activated CD8^+^ T cells. We also show that PD-L1 is initially distributed across the IS before ultimately accumulating with TCR in the center of the fully mature synapse. ICOSL, CD80 and CD86 cluster in the center of the contact area through all stages of IS maturation and colocalize with TCR in the order of ICOSL>CD86>CD80. These findings demonstrate isMap’s utility in dissecting the functional organization of the IS and highlight the dynamic redistribution of ligand-receptor pairs during T cell activation.

## 1 Introduction

The canonical IS is a radial structure composed of three primary domains – the central supramolecular activation cluster (cSMAC) dominated by peptide-MHC (pMHC) and ligated TCR, the peripheral supramolecular activation cluster (pSMAC) defined by ICAM-1 and LFA-1 and the distal supramolecular activation cluster (dSMAC) dominated by filamentous actin (F-actin) (1, 2). Additionally, a fourth domain – the CD2 corolla defined by CD58 binding to CD2 on T cells – was recently characterized (3). Costimulatory and inhibitory receptor-ligand pairs are organized within these domains in a context-dependent manner, and the IS thus serves as a signal integration platform defining the T cell response.

IS formation can be recapitulated *in vitro* by incubating T cells on glass supported lipid bilayers (SLB), a minimal component system consisting of a mobile lipid phase configured to carry proteins at calibrated densities (4). Because the synaptic interface forms directly on the substrate, this facilitates microscopy-based analysis of its molecular organization. By incorporating fluorescently tagged costimulatory and inhibitory proteins in the SLB we can thereby monitor their temporal partitioning in the various domains of the IS.

The balance between T cell costimulation and inhibition is critical for maintaining peripheral tolerance and preventing autoimmunity while maximizing protective potential (5). Among the most important modulators of T cell activation are the B7 family members CD80 and CD86. They provide a critical costimulatory signal during initial priming of naive T cells by interacting with CD28 on the T cell surface (6). TCR ligation and CD28 stimulation subsequently induces expression of ICOS, a costimulatory receptor important for the production of cytokines such as IFN-γ, IL-4, IL-10, IL-17 and to a lesser extent IL-2, upon binding to its ligand, the B7 family member ICOSL (7–9).

Following activation, T cells also upregulate inhibitory receptors to prevent hyperactivation of the immune system. One such receptor is CTLA-4 which binds CD80 and CD86 with higher affinity than CD28 and acts by removing them from the surface of APCs through trans-endocytosis (10, 11). PD-1, another inhibitory receptor upregulated on activated T cells, interacts with the B7 family member PD-L1 on APCs, triggering recruitment of the phosphatase SHP-2 which dephosphorylates TCR proximal signaling components and thereby attenuates T cell activation (12–14).

Here, we apply isMap to map the spatial organization of these immunoregulatory ligand-receptor pairs at various timepoints during T cell activation and IS formation on SLB.

## 2 Materials and methods

### 2.1 Reagents

Recombinant UCHT1 αCD3ϵ Fab with a C-terminal 12His tag was expressed in Expi 293 F cells, purified by Ni^2+^ affinity and labeled with AlexaFluor™ 488-NHS ester at ~1 fluorophore per Fab ratio. Recombinant ICAM-1-12His was generated in S9 cells, purified by Ni^2+^ affinity and labeled with AlexaFluor™ 405-NHS ester. For the other proteins, complete ECDs with a C-terminal 12His tag were expressed in 293 HEK cells and purified by Ni^2+^ affinity chromatography and gel filtration. They were labeled with Alexa Fluor™ 647-maleimide on a free cysteine added between the ECD and the 12His tag.

### 2.2 Cell culture

Peripheral blood from healthy donors was acquired from the National Health Service blood service under ethics license REC 11/H0711/7 (University of Oxford). CD8^+^ cells were isolated by negative selection (RosetteSepTM Human CD8^+^ T cell Enrichment Cocktail, STEMCELL technologies, Cambridge, UK; #15023) following the manufacturer’s protocol. Donor specific CD8^+^ T cells were plated at 1x10^6^ cells/ml with CD3/CD28 activation beads (Thermo Fisher Scientific, Loughborough, UK; #11132D) at a 1:1 ratio with 50 U/ml IL-2 at 2 ml per well in a 24 well plate and expanded for three days at 37°C in a CO_2_ incubator. On day three, cells were resuspended, counted and replenished with fresh media with 25 U/ml IL-2 to reach the original density of 1x10^6^ cells/ml. The cells were then expanded for one additional day before they were activated on SLB.

### 2.3 Planar supported lipid bilayer experiments

Flow chambers (sticky-Slide VI 0.4, Ibidi, Thistle Scientific Ltd., Glasgow, UK; #80608) were attached to cleanroom cleaned coverslips (SCHOTT UK Ltd., Stafford, UK; #1472315) and cured for 30 min. 50 ul of 2.5 mol% DOGS-NTA(Ni) lipids in DOPC was loaded in each Ibidi channel and incubated for 20 min. All lipids were obtained from Avanti Polar Lipids (Alabaster, AL). The channels were washed three times with 200 ul of 0.1% BSA/HBS (20 mM HEPES, 137 mM NaCl, 1.7 mM KCl, 0.7 mM Na2PO4, 5 mM glucose, 2 mM MgCl2, 1 mM CaCl2, pH 7.2) and blocked with 200 ul of 2% BSA/HBS for 20 min. The channels were washed again three times with 200 ul of 0.1% BSA/HBS before 200 ul of protein mix (ICAM-1-AF405, UCHT1-AF488 and either CD58-AF647, PD-L1-AF647, ICOSL-AF647, CD80-AF647 or CD86-AF647) in 0.1% BSA/HBS was loaded and incubated for 20 min before a final washing step with three times 200 ul of 0.1% BSA/HBS. All incubation steps were done at room temperature.

The protein concentrations required to reach SLB densities of 100 mol/µm2 ICAM-1, 30 mol/µm2 UCHT1 and CD58, and 1 mol/µm2 PD-L1, ICOSL, CD80 and CD86 were calculated from calibration curves determined by FCM measurements of bead supported lipid bilayers analyzed together with MESF beads (Bangs Laboratories Inc., Fishers, IN, USA) as described previously (15). The lateral mobility of the SLBs was determined by fluorescence recovery after photobleaching (FRAP) with a 10 µm bleach spot on an inverted FV1200 confocal microscope (Olympus, Tokyo, Japan).

1 x 10^5^ T cells were added to each channel and incubated at 37°C for 5, 15 or 30 min. Cells were fixed with 100 ul of 4% Paraformaldehyde (PFA) in PHEM buffer (60mM PIPES, 25mM HEPES, 10mM EGTA, and 4 mM MgSO4·7H20) for 10 min and washed 3x in PBS. TIRFM imaging was performed with an Olympus IX83 inverted microscope (Olympus, Tokyo, Japan) equipped with 405 nm, 488 nm, 561 nm and 640 nm laser lines, an Andor sCMOS Prime 95B camera (Andor Technology Ltd., Belfast, UK) and a 100x 1.45 NA oil immersion objective.

## 3 Results

### 3.1 isMap is a modular analysis tool available both as a set of Fiji macros and a Python plugin for napari

We have previously published a collection of Fiji macros designed to segment micrographs of T cells activated on SLB using a fixed threshold, measure mean fluorescence intensity across the segmented cell areas, and generate radial averaged images (16). Here, we present new versions of these original Fiji macros with added functionality, including dynamic thresholding, colocalization analysis and quantification of radial intensity, and we have developed accompanying MATLAB scripts for data visualization and plotting.

**Figure 1:**
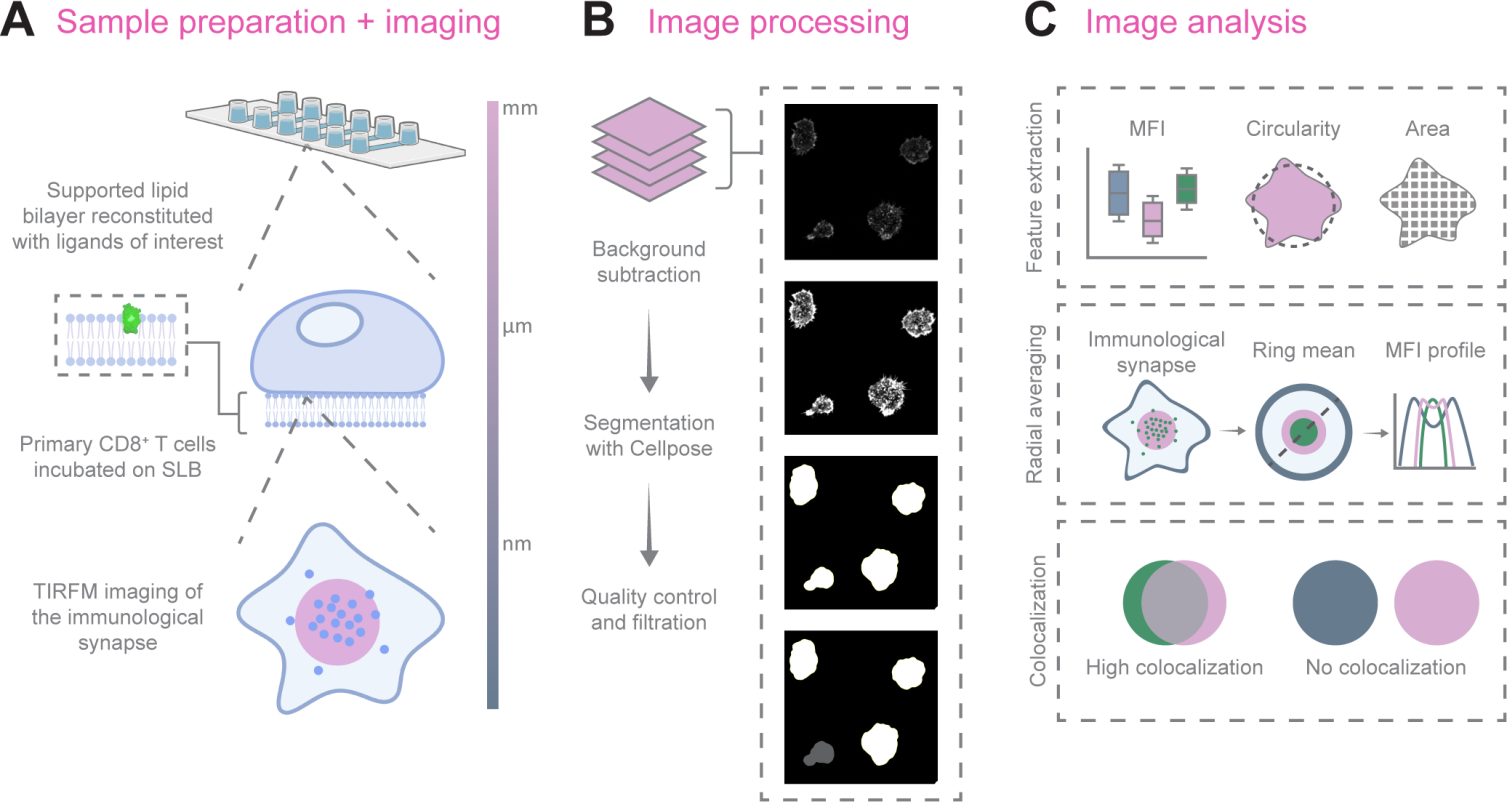
Schematic illustration of the isMap pipeline for quantitative mapping of the IS. **(A)** Images of the IS are acquired by TIRFM of human primary CD8^+^ T cells activated on SLBs reconstituted with fluorescently tagged ligands of interest. **(B)** After background subtraction, individual synapses are segmented using the integrated deep learning–based tool Cellpose. Synapses can be filtered by size and circularity to ensure consistent quality and exclude irregular or incomplete synapses. **(C)** Feature extraction quantifies key attributes such as MFI, circularity, and area, which are compiled into a single results file. The radial averaging module transforms fluorescence images into radially symmetric intensity profiles, allowing direct comparison of molecular enrichment patterns across individual synapses and experimental conditions. Pixel-wise PCCs are calculated to quantify the degree of spatial colocalization between ligands. Figure created with BioRender.com.

To further improve this tool with a streamlined workflow and improved usability, we have developed an integrated napari plugin with a graphical user interface (GUI) that removes the need for any coding by the users. Napari is an open-source, Python-based platform for viewing and analyzing multidimensional microscopy data with an active ecosystem of community-built plugins for extended functionality (17). Our new isMap napari plugin includes deep learning-based cell segmentation, filtering refinement, integrated mean fluorescence intensity (MFI) and colocalization analysis, improved radial averaging, and data visualization and plotting capability directly within the GUI (**Figure 1**). The program also outputs a JSON file with the specific analysis parameters used, thus facilitating reproducibility across experiments and users.

#### 3.1.1 Data import, segmentation and feature extraction

While napari natively supports many common microscopy formats, we extended this capability in isMap by integrating the BioIO library (18). This enables isMap to access all image formats supported by Bio-Formats, ensuring full compatibility with images from diverse microscopy vendors. As a result, users can seamlessly import and visualize virtually any microscopy dataset without requiring prior file conversion.

For cell segmentation, the isMap napari plugin uses the deep learning-based Cellpose algorithm (v3.1.1.2), employing either the cyto2 or cyto3 pretrained models (19). This eliminates the need for manual thresholding, thus providing a fast, scalable, and reproducible method for cellular segmentation across large microscopy datasets.

#### 3.1.2 Radial averaging

To systematically analyze and compare the spatial organization of the different molecules in the synapse, we developed a radial averaging module for the isMap napari plugin. The module processes 2D grayscale images of the IS and replaces each pixel with the mean intensity of all pixels located at the same integer radius from the synapse center. This produces a radially symmetric image within the bounding box of the segmented cells, from which the mean intensity is shown as a function of the distance from the center.

All radial averages belonging to the same condition or group are aggregated into a stack and the module generates three group-level outputs (1): A montage of all radial-averaged synapses for visual inspection (<Channel>_radMontage.tif) (2), a stacked TIFF file (<Channel>_radStack.tif), and (3) an overall average of all group synapses (<Channel>_radTotAv.tif).

By collapsing spatial information onto a common radial axis, this approach enables direct comparison across cells and experimental conditions. The resulting radial intensity profiles represent the enrichment of proteins within distinct regions of the IS.

Intensity profiles can be generated either with scaling, in which individual radial averages are resized to a common target length to eliminate effects of segmentation size, or without scaling, in which profile lengths vary and thus reflect absolute protein distribution across the cell population.

Overall, the radial averaging module is fast, reproducible, and provides a standardized framework to compare spatial protein distributions across cell populations.

#### 3.1.3 Pearson’s correlation coefficient based colocalization analysis

To quantify the degree of colocalization between different molecules, we added a colocalization module to isMap to calculate the PCC for each channel pair. The PCC measures the linear correlation between the pixel intensities of two images on a pixel-by-pixel basis, ranging from -1 to +1, where +1 indicates perfect positive correlation and complete colocalization, 0 indicates no linear relationship, and -1 indicates mutually exclusive localization (20). This analysis provides a quantitative measure of the degree to which two molecules occupy the same space in the IS. Higher PCC values reflect molecules that co-distribute within the same sub-synaptic region, while lower values indicate spatial segregation. Thus, this module provides an objective metric to assess the spatial colocalization between ligand-receptor pairs in the IS. However, it is important to keep in mind that colocalization does not necessarily imply interaction. Furthermore, to minimize risk of artificially inflated PCC values due to spectral crosstalk, we recommend using fluorophores with minimal spectral overlap, narrow emission filters, and sequential acquisition where possible.

### 3.2 How-to guide

Here were provide a brief how-to guide for the isMap napari plugin. Before running isMap, ensure that Python 3.11 is installed. The isMap plugin can be installed locally from GitHub or directly through the napari plugin manager.

1. Load data: Open the isMap plugin and select a parent directory containing subfolders with different conditions containing microscopy images of the immunological synapse.
2. Assign condition names: Subfolder names are used as condition labels but can be edited when prompted.
3. Set output directory: Specify the directory in which all analysis results will be stored.
4. Specify segmentation parameters: Choose the Cellpose model (cyto3 is default), set approximate IS diameter (100 pixels is default) and select a pre-interface scale between 0.3-1.0 (0.7 pixels is default). The images are resized by this scale for inference, and the diameter is adjusted automatically so the same physical size is targeted.
5. Click Run Segmentation.
6. Select segmentation condition: In the dialog window, choose which condition(s) will be used for the initial segmentation. All conditions will be segmented, but the first run allows parameter optimization before full processing.
7. Select segmentation channel: In the dialog window, select which microscopy channels to include in the analysis and specify which channel will be used for the segmentation. Channel names can be edited as needed. The initial names are automatically extracted from the image metadata.
8. Adjust filtering: Based on the initial segmentation results, adjust the diameter and circularity sliders to define cut-off ranges for filtering cells that will be included in the full analysis.
9. Click Run Analysis.
10. View and export results: isMap generates boxplots of mean channel intensities, radial averages, intensity profiles, and colocalization scores. Per-cell features for each condition, including all segmented cells and their measurements, are automatically saved in the output directory. A consolidated summary of all cells across conditions is also maintained and updates dynamically when filters are applied.

All analysis parameters used in isMap are saved in a single exportable configuration file, ensuring reproducibility.

### 3.3 isMap validation

#### 3.3.1 CD58 transiently co-localizes with CD3 before forming a corolla in the dSMAC

CD58 interacting with CD2 on CD8^+^ T cells has previously been shown to initially cluster with the TCR-CD3 complex (hereby termed CD3) in the nascent immunological synapse before segregating into a distal corolla in the dSMAC (3). To validate isMap, we therefore analyzed the synaptic organization of CD8^+^ T cells activated on SLBs presenting fluorescently tagged αCD3-Fab (UCHT1), ICAM-1 and CD58 fixed at 5, 15 and 30 min.

**Figure 2:**
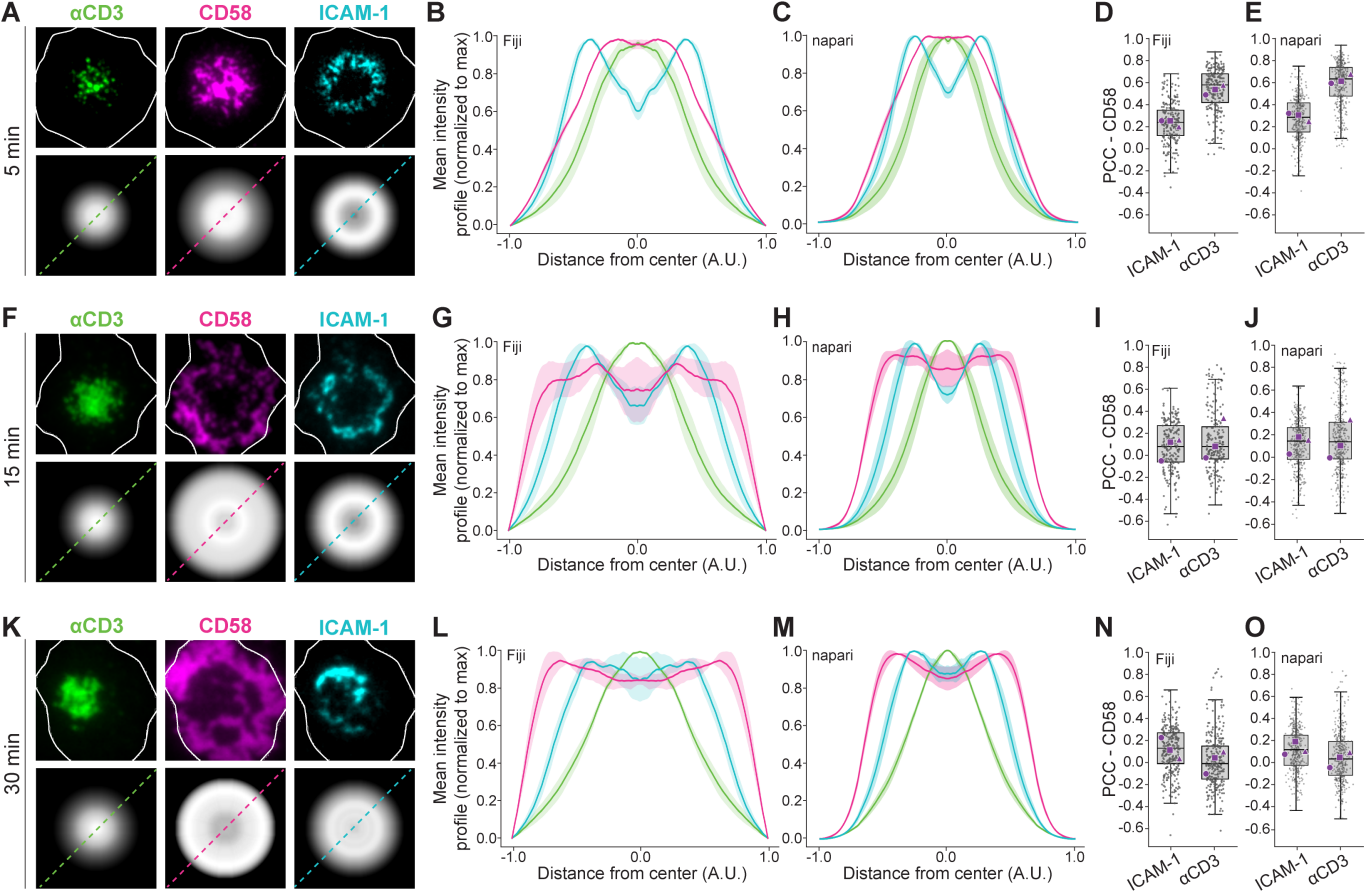
Spatial organization of CD58, αCD3 and ICAM-1 at the IS. **(A, F, K)** Representative single-cell images of synapses at 5, 15, and 30 minutes showing CD58 (magenta), αCD3 (green) and ICAM-1 (cyan). White outlines represent cell-SLB contact areas based on interference reflection images. The bottom row shows the corresponding scaled radial averaged micrographs from each channel. **(B, G, L)** and **(C, H, M)** Scaled intensity profiles (MFI ± SEM) of αCD3, ICAM-1 and CD58 from 3 independent experiments generated using Fiji and napari isMap, respectively. Y-axis intensity values are normalized to the maximum intensity of each channel. Fiji **(D, I, N)** and napari **(E, J, O)** quantification of the PCC between CD58 and ICAM-1 or αCD3 across the synaptic interface. Gray dots represent individual cells, and purple markers indicate the median from individual biological replicates. Lines denote median ± IQR. N_cells_ ≥ 200 per condition.

We observed that at 5 min, CD58 and CD3 co-distributed in the center of the contact area while ICAM-1 formed an annular ring defining the pSMAC (**Figure 2A**). We next quantified the average positional intensity of the proteins by radial averaging and confirmed their localization across the T cell population with the Fiji pipeline (**Figure 2B**) and with the isMap napari plugin (**Figure 2C**).

While the intensity profiles obtained from the two analysis tools were very similar, there were some minor discrepancies. These are likely caused by different cell segmentation approaches. In Fiji, the segmented areas are defined by a manually adjusted threshold, whereas the isMap napari plugin uses the deep learning based Cellpose algorithm and allows for parameter refinement after the initial segmentation step and prior to downstream data processing. This affects which cells are included in the analysis and the exact size of the segmented cell masks.

We next analyzed colocalization between CD3 and ICAM-1 or CD58. This correlated well with the intensity profiles as we observed PCCs between CD3 and ICAM-1 of 0.2 and 0.3 with Fiji and napari, respectively (low colocalization), and PCCs between CD3 and CD58 of 0.6 with both Fiji and napari (relatively high colocalization) (**Figures 2D, E**).

At 15 min, CD58 had segregated from CD3, and the intensity profiles indicated a redistribution across the pSMAC and the dSMAC regions (**Figures 2F–H**). However, the PCC between CD58 and both ICAM-1 and CD3 was only 0.1, indicating that CD58 traverses the pSMAC with minimal overlap with these proteins (**Figures 2I, J**).

At 30 min, CD58 had reorganized into a distal ring characteristic of the prototypical CD2-CD58 corolla as indicated by the intensity profiles showing peak CD58 intensity further from the center than peak ICAM-1 intensity (**Figures 2K–M**). This correlated with minimal colocalization between CD58 and ICAM-1 or CD3 (PCC 0.1 and 0.0, respectively) and represents the fully mature IS (**Figures 2N, O**).

#### 3.3.2 PD-L1 and ICOSL follow distinct distribution patterns during IS maturation

PD-L1 and ICOSL are both members of the B7 family and both act by modulating the immune response (5). However, whereas ICOSL mediates costimulation and induces cytokine production in T cells by binding to ICOS, PD-L1 attenuates T cell activation and induces anergy by binding to PD-1 (14, 21–25).

**Figure 3:**
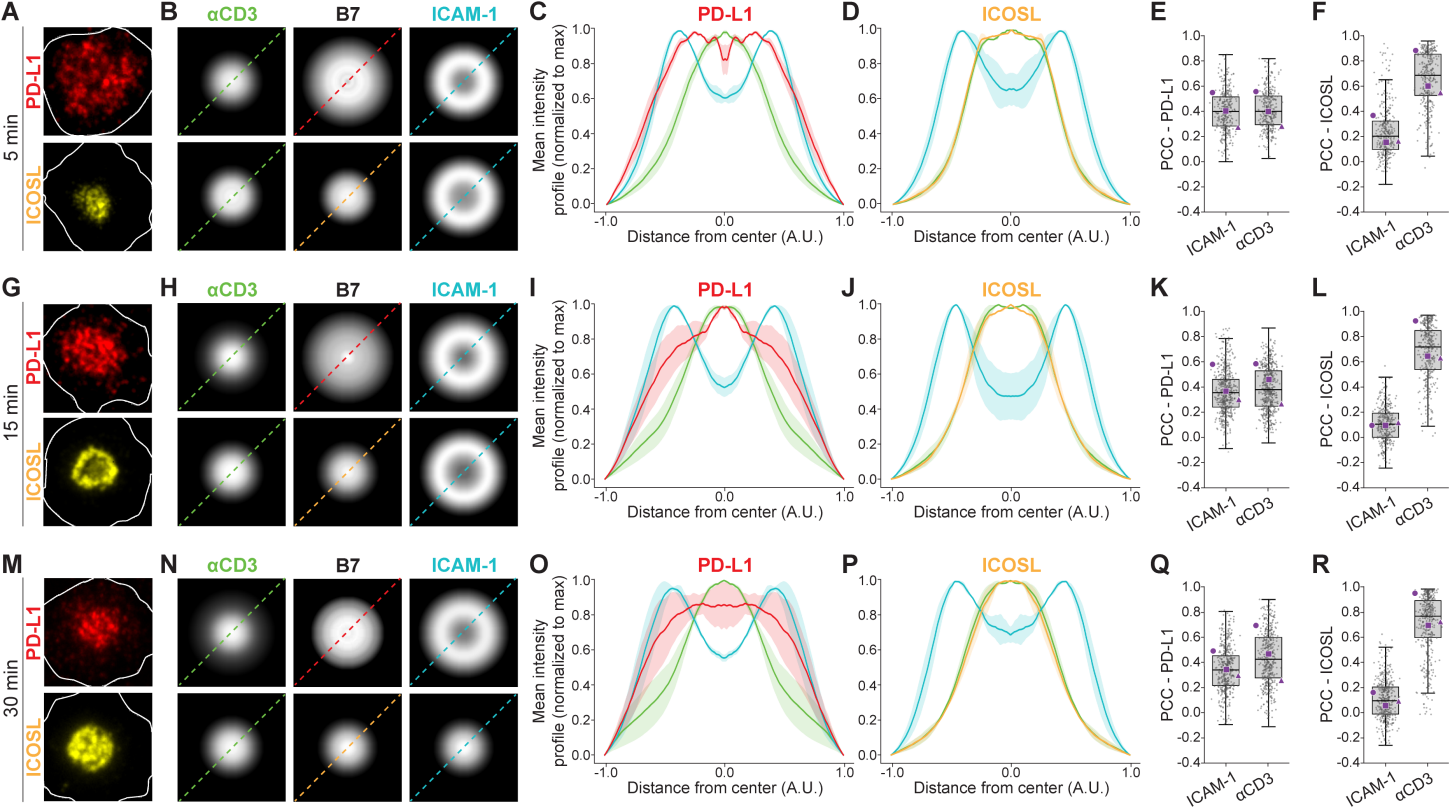
Spatial organization of PD-L1, ICOSL, αCD3, and ICAM-1 at the IS. **(A, G, M)** Representative single-cell images showing PD-L1 (red) and ICOSL (yellow) localization within the IS at 5, 15, and 30 minutes. White outlines represent cell-SLB contact areas based on interference reflection images. **(B, H, N)** Scaled radial averaged micrographs of PD-L1 (top row) and ICOSL (bottom row), denoted B7, with corresponding radial averaged micrographs of αCD3 and ICAM-1. **(C, I, O, D, J, P)** Scaled intensity profiles (MFI ± SEM) from 3 independent experiments of αCD3, ICAM-1 and PD-L1 or ICOSL with y-axis values normalized to the maximum intensity of each channel. **(E, K, Q, F, L, R)** Quantification of the PCC between either PD-L1 or ICOSL and αCD3 or ICAM-1 across the synaptic interface. Gray dots represent individual cells, and purple markers indicate the median from individual biological replicates. Lines denote median ± IQR. N_cells_ ≥ 200 per condition.

Here, we applied isMap to systematically map their synaptic distributions during CD8^+^ T cell activation on SLB as before. After 5 min, PD-L1 was distributed across the IS and colocalized with both ICAM-1 and CD3 to the same degree with PCCs of 0.4. ICOSL on the other hand, clustered in the center of the contact area and colocalized strongly with CD3 (PCC 0.7), and only marginally with ICAM-1 (PCC 0.2) (**Figures 3A–F**).

At 15 min, PD-L1 began to cluster in the center of the contact area, which was accompanied by a reduction in the colocalization between PD-L1 and ICAM-1 (PCC 0.3), while the colocalization between PD-L1 and CD3 remained at the same level (PCC 0.4). For ICOSL, the colocalization with ICAM-1 had decreased (PCC 0.1), while the colocalization with CD3 had increased slightly (PCC 0.75) (**Figures 3G–L**).

Finally, at 30 min, both PD-L1 and ICOSL clustered in the center of the contact area (**Figures 3M–P**). This correlated with a reduction in colocalization between ICAM-1 and PD-L1 (PCC 0.2), while the colocalization between PD-L1 and CD3 remained at the same level (PCC 0.4). For ICOSL, colocalization with ICAM-1 was reduced further (PCC 0.0), while colocalization between ICOSL and CD3 had again increased slightly (PCC 0.8) (**Figures 3Q, R**). This shows that the degree of overlap between both PD-L1 and CD3, and ICOSL and CD3, is relatively constant during synapse maturation, but less extensive for PD-L1 and CD3 than for ICOSL and CD3.

#### 3.3.3 Both CD80 and CD86 cluster in the cSMAC but activated CD8^+^ T cells recruit progressively more CD86 over time

**Figure 4:**
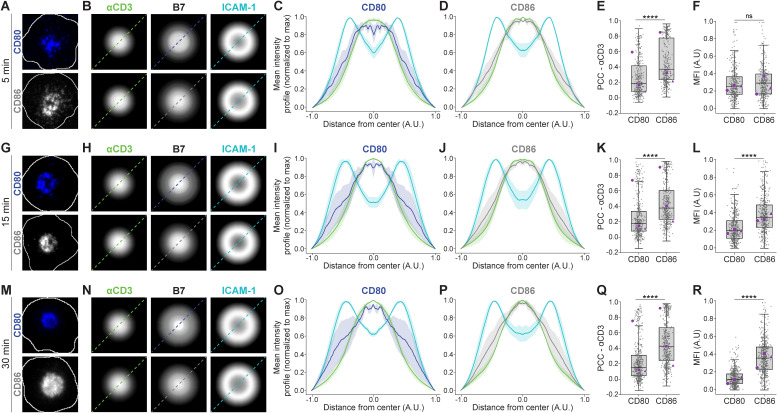
Spatial organization of CD80, CD86, αCD3, and ICAM-1 at the IS. **(A, G, M)** Representative single-cell images showing CD80 (blue) and CD86 (gray) localization within the IS at 5, 15, and 30 minutes. White outlines represent cell-SLB contact areas based on interference reflection images. **(B, H, N)** Scaled radial averaged micrographs of CD80 (top row) and CD86 (bottom row), denoted B7, with corresponding radial averaged micrographs of αCD3 and ICAM-1. **(C, I, O)** and **(D, J, P)** Scaled intensity profiles (MFI ± SEM) from 3 independent experiments of αCD3, ICAM-1 and CD80 or CD86 with y-axis values normalized to the maximum intensity of each channel. **(E, K, Q)** Quantification of the PCC between either CD80 or CD86 and αCD3 across the synaptic interface. **(F, L, R)** MFI presented as rescaled fold change within the contact area compared to the surrounding SLB. Gray dots represent individual cell values, and purple markers indicate the median from individual replicates. Lines are median ± IQR. Statistical analysis: Linear mixed model with the ligand (CD80/CD86) as a fixed effect and biological replicate as a random intercept term to account for inter-replicate variability. **** < 0.0001. N_cells_ ≥ 200 per condition.

CD80 and CD86 are critical regulators of immune activation, and they can interact both with CD28 and CTLA-4 on the surface of T cells (5). By examining their spatial organization with isMap during CD8^+^ T cell activation on SLB, we observed that both proteins accumulated in the center of the contact area already after 5 min (**Figures 4A–D**). However, colocalization with CD3 was higher for CD86 than CD80 (PCC 0.4 and 0.2, respectively) (**Figure 4E**). Note that the results are based on CD8^+^ T cells isolated from three separate donors where T cells from one of the donors displayed significantly higher colocalization between CD3 and CD80, and CD3 and CD86, than the two other donors, but that the relative difference in colocalization is similar.

As we observed differences in colocalization with CD3, we also quantified the relative amount of CD80 and CD86 recruited to the IS from the surrounding SLB, measured as MFI fold change within the T cell-SLB contact area compared to the MFI outside the contact area. However, at 5 min we could only observe a slight non-significant increase in CD86 relative to CD80 (**Figure 4F**).

At 15 min, CD80 and CD86 remained clustered in the cSMAC and colocalized with CD3 to a similar degree as before (PCC 0.2 and 0.4 for CD80 and CD86, respectively) (**Figures 4G–K**). However, the relative amount of CD86 recruited from the surrounding SLB was now 2x higher than the relative amount of CD80 (**Figure 4L**).

At 30 min, this tendency persisted, with CD80 and CD86 clustering in the cSMAC as before and colocalizing with CD3 to a similar degree (PCC 0.2 and 0.4 for CD80 and CD86, respectively), while the relative amount of CD86 recruited was now 4x higher than the relative amount of CD80 (**Figures 4M–R**).

Taken together, these results indicate that following sustained activation, CD8^+^ T cells recruit progressively more CD86 than CD80 over time.

## 4 Discussion

Here we present isMap, a modular, open-source pipeline for integrated segmentation and quantitative mapping of immune synaptic architecture. This is designed as a user-friendly general-purpose analysis tool for 2D images of fixed T cells activated on SLBs. It complements existing tools such as DeepIS (26), TIAM (27) and TIAM-HT (28), specifically designed for label-free 3D analysis of immunological synapse dynamics at T cell-APC interfaces, live 2D T cell motility analysis and high-throughput (96 well) image analysis of fixed T cells, respectively.

We show that our isMap Fiji pipeline and our napari plugin produce comparable results, while the isMap napari plugin substantially lowers the technical threshold for researchers by eliminating the need for coding expertise required in the Fiji pipeline. In addition, the configuration file and batch-processing capabilities ensure that identical analytical pipelines can be rerun or shared across laboratories.

To validate isMap, we analyzed IS formation in primary human CD8^+^ T cells activated on SLBs reconstituted with CD58, αCD3 and ICAM-1. Our results confirm that the napari plugin produces results consistent with results obtained with the Fiji pipeline and build on previous observations of dynamic molecular rearrangements during IS maturation. Consistent with previous reports, we observe that CD58 initially colocalizes with TCR microclusters in the nascent synapse before forming a corolla in the dSMAC (3).

We also apply isMap to systematically examine the temporal IS distributions of the B7 family members ICOSL, PD-L1, CD80 and CD86 in primary human CD8^+^ T cells. We show that CD80, CD86 and ICOSL cluster in the cSMAC through all stages of IS maturation, and that they colocalize with CD3 in the order of ICOSL>CD86>CD80. ICOSL presented on SLB is known to induce release of ICOS positive synaptic ectosomes (SEs) at the secretory cSMAC region of CD4^+^ T cells, while CD80, CD86 and ICOSL combined has been shown to induce release of CD28 positive SEs (15, 29). This correlates well with the cSMAC localization we observe here for these proteins.

It has previously been reported that CD80 and CD86 interact with CD28 in *cis* on the surface of activated CD8^+^ T cells, and that this interaction might compete with CD28 interactions in *trans* (30). This could explain the low levels of CD80 recruitment we observe, although the reason for the discrepancy between CD80 and CD86 recruitment is unclear. However, it has also been reported that CTLA-4 expressed on the surface of activated T cells is internalized and degraded in lysosomes upon interacting with CD80 in *trans*, while CTLA-4 interacting with CD86 dissociates from CD86 in a pH-sensitive manner and is recycled back to the plasma membrane (31). This would suggest that CD80 reduces the amount of CTLA-4 available on the T cell surface over time, possibly both by *cis* and *trans* interactions, while CD86 does not have the same effect. This could explain why we observe similar levels of CD80 and CD86 recruitment to the IS 5 minutes after T cell activation, but progressively more CD86 recruitment after 15 and 30 minutes.

Our results show that PD-L1 is initially distributed across the contact area with limited colocalization with CD3 before ultimately accumulating in the mature cSMAC. This suggests that ligated PD-1 only partially overlaps with TCR microclusters during IS maturation and thus has limited ability to affect proximal TCR signaling in the absence of additional costimulatory and/or inhibitory ligands presented on SLB. This correlates with previous reports indicating that relative PD-1 inhibition is enhanced in the presence of the CD58-CD2 corolla (3).

In summary, by combining scalable analysis with accessibility and reproducibility, isMap constitutes a powerful computational platform for examining the molecular machinery involved in T cell activation, which is illustrated by the results presented in this study. Furthermore, isMap’s modular design allows seamless integration of additional analysis modules. This flexibility ensures that isMap can evolve alongside future needs and facilitates community-driven development. In conclusion, with isMap, we have established a framework for systematic and quantitative examination of the IS and we have applied it to elucidate the spatial organization of key costimulatory and inhibitory ligands during CD8^+^ T cell activation.

## Code availability

The napari isMap plugin is available at https://github.com/aklab-Tcell-signal-integration/napari-isMap and the Fiji and MATLAB isMap scripts are available at https://github.com/aklab-Tcell-signal-integration/Fiji-isMap, including detailed user manuals. A test data set is available on Zenodo at https://zenodo.org/records/18399649.

## Data Availability

The original contributions presented in the study are included in the article/supplementary material. Further inquiries can be directed to the corresponding author.
